# Correction to: Comprehensive Analysis of ADAMTS Gene Family in Renal Clear Cell Carcinoma and ADAMTS10 Research Combining Magnetic Resonance Imaging

**DOI:** 10.1007/s12033-023-00972-z

**Published:** 2023-11-15

**Authors:** Haifeng Hu, Ying Wang, Ying Liu, Chunyu Zhang, Guoan Li, Tianyu Zhang, Bo Dong

**Affiliations:** 1 Department of Magnetic Resonance Imaging, The Second Affiliated Hospital of Qiqihar Medical College, 64 Zhonghua Xi Lu, Jianhua District, Qiqihar City, Heilongjiang Province China; 2 Department of Ultrasound, The Second Affiliated Hospital of Qiqihar Medical College, Qiqihar City, China; 3 Department of Imaging, The Second Affiliated Hospital of Qiqihar Medical College, Qiqihar City, China; 4 Department of Urology, The Second Affiliated Hospital of Qiqihar Medical College, Qiqihar City, China

**Correction to: Molecular Biotechnology** 10.1007/s12033-023-00915-8

The article “Comprehensive Analysis of ADAMTS Gene Family in Renal Clear Cell Carcinoma and ADAMTS10 Research Combining Magnetic Resonance Imaging” written by Haifeng Hu, Ying Wang, Ying Liu, Chunyu Zhang, Guoan Li, Tianyu Zhang and Bo Dong was originally published electronically on the publisher’s Internet portal (currently SpringerLink) on 20 October 2023 with open access. With the author(s)’ decision to step back from Open Choice, the copyright of the article changed on [02/11/2023] to © The Author(s), under exclusive license to The Indian Econometric Society 2023, and the article is forthwith distributed under the terms of copyright.

